# Analysis of the microbiota composition in the genital tract of infertile patients with chronic endometritis or endometrial polyps

**DOI:** 10.3389/fcimb.2023.1125640

**Published:** 2023-05-22

**Authors:** Junhua Liang, Meng Li, Lei Zhang, Yang Yang, Xia Jin, Qiongqiong Zhang, Tao Lv, Zhenyu Huang, Qinping Liao, Xiaowen Tong

**Affiliations:** ^1^ Department of Obstetrics and Gynecology, Tongji Hospital, School of Medicine, Tongji University, Shanghai, China; ^2^ Department of Obstetrics and Gynecology, Beijing Tsinghua Changgung Hospital, School of Clinical Medicine, Tsinghua University, Beijing, China

**Keywords:** microbiota composition, genital tract, infertile patients, chronic endometritis, endometrial polyps

## Abstract

**Background:**

The previous researches show that infertile patients have a higher incidence of endometritis and endometrial polyps, and the occurrence of these two diseases is related to changes in the microbiota of the genital tract. We aim to determine the composition and changing characteristics of the microbiota in the genital tract (especially the endometrium) of infertile patients with chronic endometritis or endometrial polyps, and find the correlation between it and the occurrence of diseases.

**Methods:**

This is a prospective study. We collected genital tract biopsy samples from 134 asymptomatic infertile patients receiving assisted reproductive therapy before embryo transfer. Through pathological examination and 16S ribosomal RNA(16S rRNA) sequencing, we determined the distribution of chronic endometritis and endometrial polyps in these patients, as well as their distribution of reproductive tract microorganisms.

**Results:**

Compared with the normal control group, the microbial group of reproductive tract in patients with chronic endometritis and endometrial polyps is changed, and there are significant species differences and relative abundance differences in the vagina, cervix and uterine cavity. *Lactobacillus*, the dominant flora of female genital tract, showed a change in abundance in patients with endometrial diseases. Endometrial microbiota composed of *Staphylococcus, Gardnerella, Atopobium, Streptococcus, Peptostreptococcus, Chlamydia, Fusobacterium, Acinetobacter*, etc. are related to chronic endometritis and endometrial polyps.

**Conclusion:**

The results showed that, compared with the normal control group, the endometrial microbiota of infertile patients with chronic endometritis or endometrial polyps did have significant changes in the relative abundance distribution of species, suggesting that changes in local microecology may be an important factor in the occurrence of disease, or even adverse pregnancy outcomes. The further study of endometrial microecology may provide a new opportunity to further improve the diagnosis and treatment strategy of chronic endometritis.

## Introduction

Chronic endometritis (CE) is a disease in which the endometrium is infected and the inflammatory reaction persists. Because of its atypical clinical symptoms, inconsistent diagnostic methods, and inappropriately targeted treatment, it is still a gynecological disease that is underestimated and ignored clinically. A survey on the understanding and management of CE by obstetricians and gynecologists in the United States shows that doctors have a series of worrying defects in the pathophysiology, clinical manifestations and diagnostic methods of CE, especially in the treatment of CE ([[NoAuthor]]). Because CE has no typical clinical manifestations and is difficult to diagnose, the actual prevalence of CE in the general population is not clear and fluctuates widely, estimated to be between 0.8% and 25.2%. Although there is no typical symptom free clinic, studies have found that CE will reduce the success rate of natural and assisted reproductive technology (ART) successful pregnancy, and lead to obstetric and neonatal complications (Miles et al., 2017). In addition, among women diagnosed with unexplained repeated abortion or repeated implantation failure, the CE incidence rate was as high as 60% or 66%, respectively (Kimura et al., 2019). Therefore, it is particularly important to deeply explore the characteristics of CE pathogenic microorganisms and explore effective diagnosis and treatment methods. In the past, it was generally believed that endometrial polyp (EP) was an inflammatory disease, or a manifestation of chronic endometritis, which was formed by the reactive proliferation of endometrium under long-term sustained mechanical stimulation and biological inflammatory factors. However, recent studies have shown that the occurrence of EPs may involve many factors, such as the expression imbalance of estrogen receptor (ER) and progesterone receptor (PR), long-term sustained high level estrogen stimulation, abnormal cell apoptosis and proliferation, gene mutation, local endometrial tissue stimulated by inflammation, endometrial cell oxidative stress, etc. Therefore, this study included EP into the study separately according to the postoperative pathological results.

In recent years, the Human Microbiome Project (HMP) has found that about 9% of human microbiota exists in female genital tract (Margulies et al., 2022). In the past, it was believed that female genital tract microorganisms only inhabit the vagina, vulva, anus and so on. Because of the existence of the cervix and cervical mucus plug, the vagina and the upper genital tract are perfectly isolated, and the sterile state of the uterine cavity is maintained. However, more and more evidence shows that the female genital tract is an open system. From the external genitalia to the internal genitalia, there is a gradually changing microbiota continuum. The bacterial abundance from vagina to ovary decreases, while the bacterial diversity increases (Peterson et al., 2009; Cicinelli et al., 2015; Kitaya et al., 2015). It is reported that there are 10^2^-10^4^ fewer bacteria in the uterine cavity than in the vaginal microbiota ([[NoAuthor]]). Therefore, the uterine cavity contains a low abundance of bacterial communities, also known as low biomass microbiota. These low abundance microbiota constitute the intrauterine microecology. Many studies, including HMP, have found the importance of microorganisms and their genomes in human health and disease (Chen et al., 2017; Margulies et al., 2022). So we boldly speculate that chronic endometritis (CE), endometrial polyps (EP), and even endometrial cancer may occur when the microbiota in the uterus changes or pathogens invade. The study found that among many endometrial diseases, CE and EP are more common in patients with infertility and become the main factors affecting the outcome of assisted reproduction (Human Microbiome Project Consortium, 2012; Miles et al., 2017; Peric et al., 2019).

By using 16S ribosomal RNA(16S rRNA) sequencing technology, this study investigated and analyzed the vaginal, cervical and endometrial biopsy (EB) samples of 134 infertile patients who were ready for assisted reproductive technology (ART), and took endometrial tissue for pathological examination. The main purpose of this study is to further explore the genital tract microecological characteristics of infertile patients with CE and EP by analyzing the germs (especially the upper genital tract) of asymptomatic infertile patients, and lay a foundation for finding the pathogenesis of the disease and effective diagnosis and treatment methods.

## Methods

### Study design and study population selection

In this prospective study, we analyzed vaginal microbiome (VM), cervical microbiome (CM), and endometrial microbiome (EM) in infertile patients prepared for ART treatment by 16S rRNA sequencing. At the same time, endometrial tissue was obtained during hysteroscopy for pathological examination and immunohistochemical examination, and pathological diagnosis was made according to the current CE and EP pathological diagnostic criteria, which served as the basis for grouping.

The main purpose of this study is to conduct a preliminary study on the characteristics of the genital tract microbiota of CE patients, especially the endometrial microbiota, and to study the possible pathogenesis of CE by comparing the microbiota of the genital tract in CE, EP and normal control groups.

In this study, infertile patients who are ready to receive assisted reproductive technology are selected as the research object, mainly based on the following considerations: First, according to the existing research data, the CE incidence of infertile patients is higher than that of other populations. As a prospective study, it is easier to obtain positive patients; Second, the hormone levels of patients who are ready to receive ART have been adjusted to an average level before embryo transfer, minimizing the impact of hormones on the genital tract microecology.

### Study population—inclusion and exclusion criteria

Select the patients who are scheduled to undergo hysteroscopy before ART in our hospital, since October 2020 and meet the inclusion criteria, and take the genital tract microbial samples and endometrial tissue samples of the patients during hysteroscopy.

Inclusive criteria were: age>18 years and<45 years; within 3-5 days of clean menstruation (excluding abnormal vaginal bleeding); the serological tests of human immunodeficiency virus, hepatitis B, hepatitis C virus and syphilis were negative; the vaginal discharge was normal before operation; no antibiotics were taken within 30 days before operation; no sexual life within 3 days before operation; there was no vaginal irrigation or medication within 7 days before operation.

Exclusion criteria were: acute stage of various female genital tract inflammation; systemic acute inflammation; patients with autoimmune diseases; intrauterine placement of birth control ring; there are uterine cavity operators within 3 months.

### Sample collection

All samples in this study were collected during hysteroscopic surgery: 1) Vaginal secretions were collected with sterile cotton swabs before sterilization and placed in sterile PBS; 2) Specimens of cervical secretion: after routine surgical disinfection, expose the cervix, place the cervical microbial sampling brush in the cervical tube and rotate it for 10 circles, collect the cervical secretion samples and put them in sterile PBS; 3) Specimens of uterine secretion: insert the special endometrial sampling brush with a sleeve into the uterine cavity, withdraw the sleeve, expose the brush head and rotate for 10 circles to collect samples of uterine secretion, push back the sleeve, take out the endometrial sampling brush, and place the sample in sterile PBS. 4) Endometrial tissue samples: after inserting the hysteroscope according to the routine operation of hysteroscopy, observe and record the condition of the endometrium. At the same time, gently scratch the uterine cavity with a small curette for one week, and collect endometrial tissue samples. After all samples are taken, samples of vaginal secretions, cervical canal secretions and uterine cavity secretions shall be immediately stored in a refrigerator at - 80 °C and sent to the laboratory for 16S rRNA sequencing analysis. Endometrial tissue samples were placed in sterile formalin solution and sent for pathological examination.

### Pathological diagnosis and grouping

The diagnosis and grouping of all enrolled patients were determined based on the pathological examination results of endometrial tissue samples. All patients with pathological diagnosis of “chronic endometritis” or “chronic inflammation of the endometrium with extensive lymphocyte infiltration”, and at least one positive item of CD38 or CD138 in immunohistochemical examination results, are included in the CE group. Patients with pathological findings of “endometrial polyps” or “endometrial polypoid hyperplasia” were included in the EP group. The other patients without abnormal pathological diagnosis were all included in the normal control group, namely NE group. According to the above grouping, the corresponding 16S rRNA sequencing results were statistically analyzed.

### Sequencing

1. Extraction of genome DNA: Total genome DNA from samples was extracted using CTAB method. DNA concentration and purity was monitored on 1% agarose gels. According to the concentration, DNA was diluted to 1ng/µL using sterile water.

2. Amplicon Generation: 16S rRNA genes of distinct regions(16S V4/16S V3/16S V3-V4/16S V4-V5) were amplified used specific primer(e.g. 16S V4: 515F 806R, et. al) with the barcode. All PCR reactions were carried out with 15 µL of Phusion^®^ High-Fidelity PCR Master Mix (New England Biolabs); 2 µM of forward and reverse primers, and about 10 ng template DNA. Thermal cycling consisted of initial denaturation at 98°C for 1 min, followed by 30 cycles of denaturation at 98°C for 10 s, annealing at 50°C for 30 s, and elongation at 72°C for 30 s. Finally 72°C for 5 min.

3. PCR Products quantification and qualification: Mix same volume of 1X loading buffer (contained SYB green) with PCR products and operate electrophoresis on 2% agarose gel for detection. PCR products was mixed in equidensity ratios. Then, mixture PCR products was purified with Qiagen Gel Extraction Kit(Qiagen, Germany).

4. Library preparation and sequencing:Sequencing libraries were generated usingTruSeq^®^ DNA PCR-Free Sample Preparation Kit (Illumina, USA) following manufacturer’s recommendations and index codes were added. The library quality was assessed on the Qubit@ 2.0 Fluorometer (Thermo Scientific) and Agilent Bioanalyzer 2100 system. At last, the library was sequenced on an Illumina NovaSeq platform and 250 bp paired-end reads were generated.

### Data analysis

Raw data obtained from sequencing has a certain proportion of Dirty Data. In order to make the results of information analysis more accurate and reliable, the original data shall be spliced and filtered to obtain valid data (Clean Data).

Based on the effective data, OTUs (Operational Taxonomic Units) clustering and species classification analysis were carried out. According to the OTUs clustering results, on the one hand, species annotation was made for the representative sequence of each OTU to obtain the corresponding species information and the abundance distribution based on species. At the same time, OTUs were analyzed by abundance, Alpha diversity calculation, Venn map and petal map to obtain the species richness and evenness information in the sample, and the common and unique OTUs information among different samples or groups. On the other hand, multiple sequence alignment can be carried out for OTUs, and phylogenetic trees can be constructed, and further differences in community structure of different samples and groups can be obtained, which can be displayed through dimension reduction maps such as Beta diversity calculation, PCoA or PCA.

In order to further explore the differences in community structure among grouped samples, T-test, MetaStat, LEfSe and MRPP statistical analysis methods were used to test the difference significance of species composition and community results of grouped samples.

## Results

### Patient cohort, characteristics, and outcomes

From October 2020 to July 2021, 169 infertile patients in our department who are ready to receive IVF treatment were evaluated. Twenty nine patients were excluded from the study because they did not meet the inclusion criteria (n=29) or refused to participate (n=4). The remaining 134 people were enrolled in the study, and the composition of microbiota in genital tract of patients with different pathological types was evaluated by 16S rRNA sequencing and pathological examination. Among them, there were 29 CEs (21.6%), 20 EPs (14.9%), and 85 normal endometrium (showing normal secretory or proliferative endometrium) (63.5%). The average age of the patients was 33 years (33 ± 7.6years). There was no significant difference in general clinical data between groups.

### General situation of genital tract microbiota composition

Due to the low abundance of microbiota in the endometrium, we performed a rigorous analysis of the sample data to ensure that contamination readings did not interfere with downstream analysis. The undetectable samples are excluded by comparing the samples with the samples in each run (including blank samples) and evaluating certain quality parameters.

**Figure 1:**
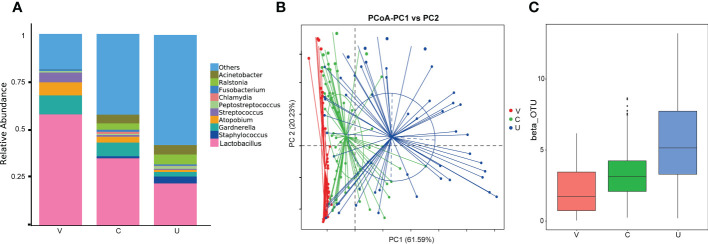
**(A)** Histogram of Relative Abundance Distribution of Microbes in Patient’s Genital Tract. List the top ten microorganisms in relative abundance on genus level and others. Group name: V= vaginal microbiome, C=cervical microbiome, and U=endometrial microbiome. **(B, C)** Comparative analysis of microbial community composition in genital tract. PoCA shows clustering between groups of samples. Beta diversity index box chart and table visually shows significant differences between different groups. Group name: V= vaginal microbiome, C=cervical microbiome, and U=endometrial microbiome.

All detectable samples were included in the analysis according to the above standards, and the overall analysis results of the samples showed that: *Lactobacillus* is the main genus in all samples. At the same time, bacterial genera (such as *Acinetobacter, Atopobium, Fusobacterium, Gardnerella, Peptostreptococcus, Staphylococcus, Streptococcus* and *Streptococcus*) and *Chlamydia* are also common. All genital tract microbial samples are grouped according to vagina, cervix and endometrium, and the relative abundance and distribution of species in each group are shown in **Figure 1A**. Statistical analysis shows that the abundance of the same genus in different parts is significantly different (and **Figures 1B, C**).

From **Figures 1B, C**, we can find that at the genus level, the relative abundance of *Lactobacillus, Gardnerella, Atomium* and *Streptococcus*, decreases gradually from the lower genital tract to the upper genital tract, while that of *Staphylococcus, Ralstonia* and *Acinetobacter*, including others, increases gradually. *Chlamydia* and *Fusobacterium* have high abundance in the cervix.

### Taxonomic analysis of species in genital tract of CE and EP patients

At the same time, we also divided the patients into CE group, EP group and normal endometrium group(NE) according to the pathological examination results, and on the level of phylum, class, order, family, genus and species, the composition of microbiota in different parts of genital tract of patients with different pathological results was analyzed.

### OTU cluster analysis

We use software to cluster the sequence data obtained at 97% similarity level to get OUT. According to the clustering results, we analyzed the common and unique OTUs among different sample groups (**Table 1**)

**Table 1:** Number of OTUs in genital tract specimens of patients in each group.

Group	Number of OTUs	Subtotal	Total
**CEV**	441	5979	17685
**CEC**	3274		
**CEU**	2264		
**PV**	316	2550	
**PC**	1103		
**PU**	1131		
**NV**	843	9156	
**NC**	5397		
**NU**	2916		

**Figure 2:**
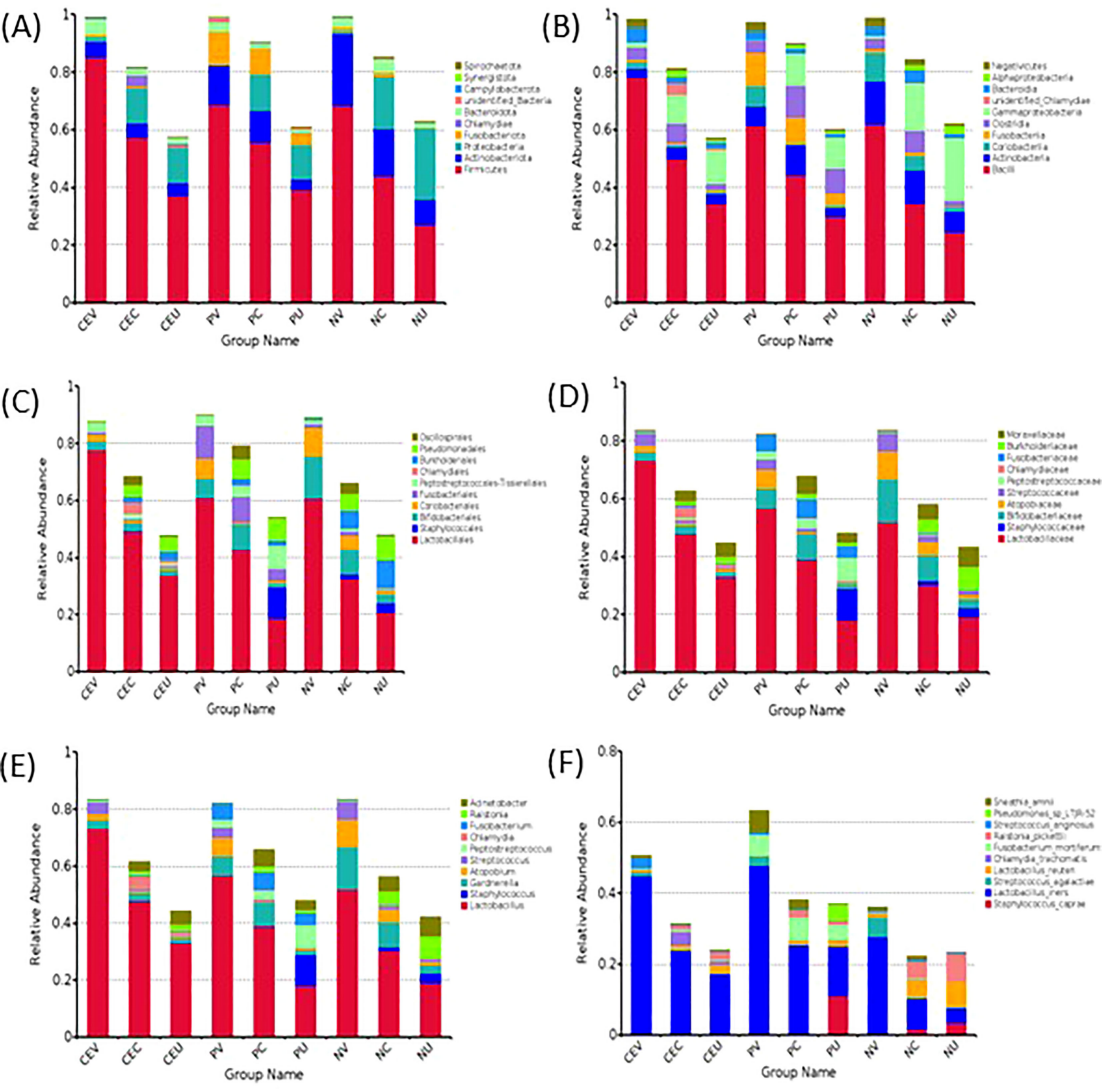
Histogram of Relative Abundance Distribution of Microbes in Patient’s Genital Tract. List the top ten microorganisms in relative abundance on phyla level **(A)**, class level **(B)**, order level **(C)**, family level **(D)**, genus level **(E)** and species level **(F)** without others. Group name: CEV=CE patients’ vaginal microbiome, CEC=CE patients’ cervical microbiome, and CEU= CE patients’ endometrial microbiome; PV=EP patients’ vaginal microbiome, PC= EP patients’ cervical microbiome, and PU=EP patients’ endometrial microbiome; NV= vaginal microbiome in patients with normal endometrium, NC=cervical microbiome in patients with normal endometrium, and NU=endometrial microbiome in patients with normal endometrium.

Group name: same as **Figure 2**.

### Taxonomic analysis of species

Complete the OUT clustering and annotate the OTUs sequence. According to the species annotation results, select the top 10 species with the highest abundance on each classification level (Phylum, Class, Order, Family, Genus, Species) for each grouping, and generate a histogram of species relative abundance (**Figures 2A–F**) . At the same time, the composition ratio of the patient’s genital tract microbiota on each level is generated. Because the distribution of the microbiota at each level is highly consistent, only the composition ratio tables at the phyla level and genus level are listed in this paper (**Tables 2**, **3**).

**Table 2:** Composition ratio of patients’ genital tract microbiota on phylum level.

Taxonomy	NV	CEV	PV	NC	CEC	PC	NU	CEU	PU	
Firmicutes	0.684	0.851	0.686	0.438	0.572	0.555	0.269	0.372	0.390	
Actinobacteriota	0.250	0.055	0.136	0.165	0.051	0.113	0.090	0.045	0.039	
Proteobacteria	0.010	0.017	0.002	0.182	0.122	0.124	0.246	0.121	0.118	
Fusobacteriota	0.012	0.008	0.116	0.014	0.007	0.090	0.002	0.005	0.043	
Chlamydiae	0.000	0.000	0.000	0.001	0.039	0.001	0.000	0.010	0.000	
Bacteroidota	0.032	0.054	0.032	0.045	0.023	0.017	0.015	0.019	0.012	
unidentified_Bacteria	0.004	0.002	0.018	0.004	0.002	0.003	0.004	0.002	0.005	
Campylobacterota	0.001	0.004	0.001	0.003	0.001	0.002	0.002	0.001	0.000	
Synergistota	0.000	0.000	0.000	0.000	0.000	0.000	0.001	0.000	0.000	
Spirochaetota	0.000	0.000	0.000	0.000	0.000	0.000	0.000	0.000	0.002	

Group name: same as **Figure 2**.

List the top 10 microorganisms in relative abundance on phyla level.

**Table 3:** Composition ratio of patients’ genital tract microbiota on genus level.

Taxonomy	NV	CEV	PV	NC	CEC	PC	NU	CEU	PU
Lactobacillus	0.519	0.732	0.567	0.301	0.477	0.388	0.190	0.330	0.181
Staphylococcus	0.001	0.000	0.000	0.016	0.003	0.003	0.034	0.001	0.110
Gardnerella	0.148	0.030	0.067	0.087	0.028	0.082	0.028	0.017	0.016
Atopobium	0.096	0.023	0.066	0.047	0.007	0.005	0.015	0.007	0.007
Streptococcus	0.063	0.041	0.036	0.010	0.008	0.006	0.007	0.004	0.002
Peptostreptococcus	0.005	0.006	0.027	0.003	0.004	0.032	0.002	0.002	0.079
Chlamydia	0.000	0.000	0.000	0.001	0.039	0.001	0.000	0.010	0.000
Fusobacterium	0.002	0.002	0.058	0.003	0.005	0.064	0.001	0.003	0.042
Ralstonia	0.000	0.000	0.000	0.046	0.013	0.019	0.077	0.023	0.012
Acinetobacter	0.000	0.000	0.000	0.051	0.033	0.059	0.068	0.046	0.031

List the top 10 microorganisms in relative abundance on genus level. Group name: same as **Figure 2**.

As can be seen from the **Figure 2A**, the top 10 on phyla level include *Firmicutes, Actinobacteriota, Proteobacteria, Fusobacteriota, Chlamydiae, Bacteroidota, Campylobacterota, Synergistota, Spirochaetota* and unidentified_Bacteria.

Compared with NE group, among the top five phylums in CE group, the distribution of *Firmicutes* in the whole genital tract increased significantly. On the contrary, *Actinobaciota*, which ranked second, showed a decreasing trend. The distribution of *Proteobacteria* in endometrium was significantly reduced. *Fusobacteriota* decreased in vagina and cervix, but increased in uterine cavity. *Chlamydiae* is almost not detected in the vagina and uterine cavity, but it is detected in the cervix, and the abundance of CE patients is significantly increased. However, in NP group, the distribution trend of *Firmicutes* and *Actinobaciota* in the whole genital tract was the same as that in CE group. *Proteobacteria* showed a decreasing trend in the distribution of the whole genital tract. *Fusobacteriota* increased in the whole genital tract. *Chlamydiae* was not detected in the genital tract of NP patients.

At the class level, the top 10 classes mainly come from the top 5 phylums. They are mainly Bacilli, Clostridia, Negativicutes from Firmicutes, and Actinobacteria, Coriobacteriia from Actinobacteria. Compared with NE group, the distribution change of relative abundance of microbiota in CE group and NP group was consistent with the change of phylum level.

Through statistical analysis, we also found that at the level of orders, families, genera and species, the microbial species with higher relative abundance also mainly come from the top five phyla, and the species with higher relative abundance and their proportion increase or decrease are consistent with the level of phyla and class.

### Species abundance clustering heat map

**Figure 3:**
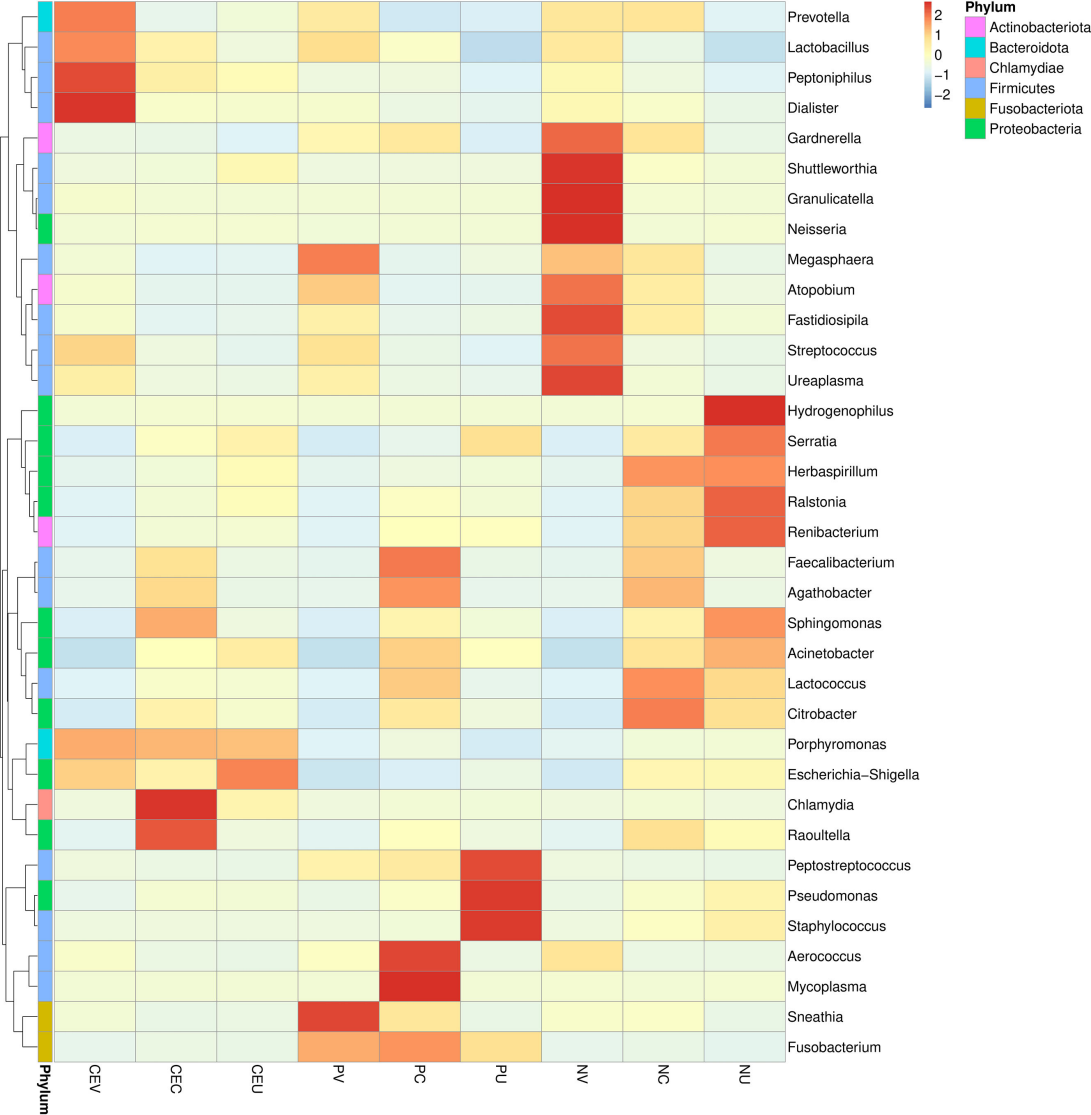
Cluster heat map of microbial species abundance in genital tract of patients in each group. In the figure, the horizontal is the sample information of each group, the right side is the species annotation information, and the left side is the species clustering tree. The color of each grid indicates the abundance of species. Group name: same as **Figure 2**.

In order to further clarify the difference in relative abundance of microorganisms among groups and find out the distribution characteristics of genital tract flora of CE and NP patients, according to the species annotation and abundance information of all samples on the genus level, the top 35 genera with abundance ranking are selected. According to their abundance information, they are clustered from two different levels, namely species and grouping, and drawn into a heat map, so as to more intuitively find the species that gather more or less in each group, as well as the differences between groups (**Figure 3**).

### Alpha diversity analysis

**Figure 4:**
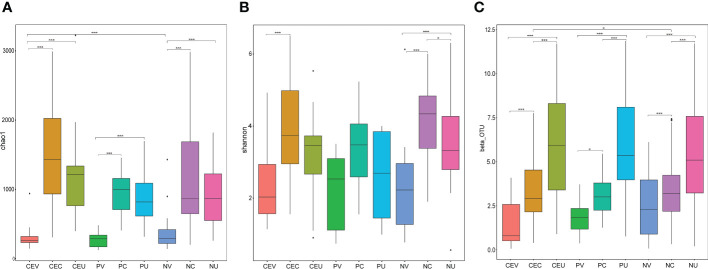
Box Chart of Chao1 **(A)**, Shannon **(B)** Index and Beta Diversity **(C)** of Genital Tract Microbial Samples of Patients in Each Group. Group name: same as **Figure 2**.

In order to evaluate the differences in species richness and diversity of microbial communities in various groups, we made statistics on the alpha diversity analysis index (chao1, shannon) of different samples under the 97% consistency threshold. At the same time, analyze whether the species diversity difference between groups is significant by Wilcox rank sum test (**Figures 4A, B**).

### Beta diversity analysis

In order to compare the difference of microbial community composition of different sample groups, we made statistics on the beta diversity analysis. And, analyze whether the species composition difference between groups is significant by Wilcox rank sum test (**Figure 4C**).

## Discussion

Chronic endometritis (CE) is a chronic inflammatory disease with endometrial interstitial plasma cell infiltration, which is mainly caused by changes in endometrial microecology and infection of pathogens (Chen et al., 2017; Moreno and Cicinelli, 2018). And endometrial polyps (EPs) are a kind of pathological changes that protrude from the surface of endometrium, which are composed of endometrial glands and fibrotic endometrial stroma containing blood vessels (Nijkang et al., 2019). The main purpose of this study is to understand the composition and change characteristics of the microbiota in the genital tract (especially in the endometrium) of infertile patients with CE or EPs, and to explore its correlation with disease occurrence. Some diseases or organ dysfunction are characterized by the presence of potentially pathogenic microorganisms. Research based on molecular sequencing technology shows that the microbiota of female genital tract is in dynamic balance under healthy conditions, and the change of microbiota composition of genital tract may be related to a variety of gynecological diseases, such as chronic endometritis (CE), endometrial polyps (Eps), endometriosis (EMs), endometrial cancer (EC), infertility, etc (Al-Nasiry et al., 2020). From **Figures 1A–C**, and other results, we can easily find that the microorganisms in the genital tract show the characteristics of continuous distribution, and the species abundance in different parts of the genital tract has a significant difference, indicating that different parts of the genital tract have different microbial distribution characteristics, in other words, they have different micro ecological environments. When local microbial status changes or pathogenic bacteria invade, corresponding dysfunction or disease will occur.

### About the OTUs

In the OUT cluster analysis, we found that, according to the location, the number of species from vagina to uterine cavity increased gradually. According to disease grouping, the number of genital tract species in NE group was significantly higher than that in CE and NP groups, and the number of species in CE group was higher than that in NP group.

This result indicates that the abundance of microorganisms gradually decreases from the lower genital tract to the upper genital tract, but the number of species gradually increases. Similar findings have also been made in previous studies. Mile et al (Miles et al., 2017). evaluated the distribution of bacteria in the entire female genital tract, including the fallopian tubes and ovaries, and found that bacteria colonized the entire female genital tract, and the microbial community at each anatomical site was highly correlated. Chen et al. (2017) also found that bacterial abundance gradually decreased from lower to upper in the genital organs. Different bacterial communities were found in the cervical canal, uterus, fallopian tubes, and abdominal fluid compared to the vagina, and the bacterial composition in the endometrium was significantly different from that in the lower genital tract.

Fang et al. (2016) found that there were significant differences in the uterine microbiota between CE group patients and normal individuals, and the normal control group had a more diverse intrauterine microbiota than CE group patients. This is similar to our research results, indicating that the normal population has the most abundant microbial species in the genital tract, which is necessary to maintain a healthy microecology.

### About diversity analysis

whether in Alpha diversity analysis or Beta diversity analysis, we found that there were some differences between groups among microbial samples from the same genital tract in different disease groups, but these differences were not statistically significant. It indicates that there is no significant difference between CE or NPs patients and NE patients in terms of microbial species diversity or microbiota composition. Therefore, it can be inferred that the changes of local microecology of genital tract in patients with CE or Eps are mainly due to the invasion of pathogenic bacteria and the changes of the abundance of symbiotic bacteria *in situ*, and the occurrence of disease is more closely related to the changes of the abundance of bacteria.

### Changes in the composition of vaginal microbiota in patients

In a healthy state, the vaginal microbiota is in a dynamic equilibrium state dominated by *Lactobacillus*, which changes with menstrual cycle, age, pregnancy, sexual behavior, environment and other factors (Champer et al., 2018). When the composition of the lower genital tract microbiota changes, the risk of uterine cavity colonization caused by bacteria ascending increases, which can induce uterine cavity microecology disorder, and then lead to endometrial lesions. Marchenco et al (Marchenko et al., 2016). also found that the risk of vaginal bacteria colonization in patients with vaginal microecology disorder was 3.5 times higher than that in healthy women. Kovalenko et al. (2009) also proposed that *Gardnerella vaginalis* associated with BV can promote the occurrence of EPs.

From the results of species classification analysis (**Figure 2**, **Tables 2**, **3**), it can be found that compared with the EN group, the CE group and NP group had significant changes in the vaginal microbiota abundance. The main manifestation was that the abundance of dominant *Lactobacillus* was significantly increased, especially in the CE group. The abundance of *Gardnerella, Atopobium and Streptococcus* in the two disease groups decreased significantly. It can be seen more intuitively from the Species isolation clustering heat map that the distribution area of the dominant and inferior vaginal bacteria in CE group and NP group is completely different from that in NE group. Therefore, it is reasonable to infer that the changes in the composition of vaginal microbiota in patients with CE and NPs are related to the occurrence of diseases. It is possible that changes in the microbiota of the lower genital tract have led to an increased susceptibility to inflammation in the lower genital tract, increasing the possibility of certain bacteria retrograde to the upper genital tract, thereby altering the distribution of bacteria in the upper genital tract and altering local microbiota, resulting in an increased susceptibility to inflammation in the upper genital tract. Some studies this year also suggest that dysbiosis of the uterine microbiota may be one of the important promoting factors for the formation of CE and EP.

### Changes in the composition of cervical microbiota in patients

In the past, few independent studies were conducted on cervical microbiota, and most of them were conducted jointly with vaginal microbiota. Relevant studies have shown that *Lactobacillus* is the dominant bacteria in the cervical microbiota of healthy women, and other common bacteria are *Prevotella, Streptococcus and Fusobacterium*, etc (Ata et al., 2019). At the same time, Chen et al (Kimura et al., 2019). further studied and found that the proportion of *Lactobacillus* in cervical microbiota is generally slightly lower than that in vaginal microbiota.

In this study, the cervix was studied as an independent group. Our study also found that the dominant microorganism in the cervix is *Lactobacillus*, and its overall abundance is lower than that in the vagina, but higher than that in the uterine cavity. In the cluster heat map, it was found that the cervical microorganisms in NP group were mainly *Lactobacillus* and *Citrobacter*, but there was no obvious aggregation compared with other species, and the abundance of each species was relatively average. However, in CE group and NP group, the species aggregation is obvious, and the dominant bacteria are significantly different from NE group. The dominant bacteria in CE group are *Chlamydia* and *Raoultella*, while the dominant bacteria in NP group are *Mycoplasma* and *Faecalibacterium*. In the OTU cluster analysis, we found that the number of microorganism species in the cervix of CE group and NE group was significantly higher than that in the uterine cavity. The analysis may be due to the influence of some microorganism species in the uterine cavity due to the deep sampling depth of the cervix.

### Changes in the composition of intrauterine microbiota in patients

Previous studies found that the composition changes of the upper genital tract microbiota in CE and EPs patients were mainly due to the increase in the detection rate of vaginal bacteria (such as *Lactobacillus*) in the uterine cavity, while the related studies on the changes of the composition of the oviduct and ovary microbiota have not been reported. Cicinelli et al. (2009) showed that compared with the healthy control group (6.2%), the detection rate of *Lactobacillus* uteri in EPs and CE patients was higher (38.6% and 33.2% respectively). Fang et al. (2016) studied endometrial specimens of patients in the simple polyp (EP) group and the EP/CE group, and found that the intrauterine bacterial flora of patients in the EP/CE group was more diverse than that in the simple EP group and the healthy control group. *Lactobacillus, Gardnerella, Bifidobacterium, Streptococcus* and *Argyromonas* in endometrium of patients in EP/CE group and EP group were significantly increased, while *Pseudomonas* was significantly decreased. In addition, the proportion of *Enterobacteriaceae* and *Sphingolimus* in endometrium of patients in EP/CE group was decreased, and *Prevotella* was increased. It is suggested that there may be an increase of vaginal bacteria in the uterine cavity of EP/CE patients, and there is a difference in the uterine microbiota between EP/CE patients and normal people, but the relationship and mechanism are still uncertain.

In this study, we found that the lactobacillus abundance in CE group was significantly higher than that in NE group, but decreased in NP group. More interestingly, *Gardnerella* and *Atopobium*, which were found to have significantly increased abundance in other studies, were found to be significantly lower than the control group in this study. At present, the conclusions of studies on the composition and abundance of uterine microbiota are different, and further studies on the changes of specific microbial composition and pathogenic microorganisms in endometrium are still needed.

According to analysis, the bacteria species with obvious aggregation in NE group endometrium decreased significantly in CE group and NP group. On the contrary, the bacteria gathered in the two disease groups had lower content in NE group. In addition, it was also found that there was no obvious aggregation of microorganisms in the endometrium of CE group, and the concentration of each bacterium was relatively uniform, only *Porphyromonas* and *Escherichia* showed a certain degree of aggregation, and it was found that the concentrations of these two bacteria were also high in the genital tract under CE. In NP group, the intrauterine concentrations of *Peptostreptococcus, Pseudomonas* and *Staphylococcus* were significantly increased, and *Staphylococcus*, as a common pathogenic bacterium, only had a high abundance in the inner membrane of NP group. *Porphyromonas* and *Escherichia* increased in CE group, but decreased significantly in NP group. It is suggested that the occurrence of CE, NPs and other endometrial diseases is related to the reduction of normal flora. *Porphyromonas* and *Escherichia* are highly correlated with the occurrence of CE, while *Peptostreptococcus, Pseudomonas and Staphylococcus* are more correlated with the occurrence of NPs.

### About mycoplasma and chlamydia

In our study, we found that the overall abundance of chlamydia was significantly higher than that of mycoplasma, and the highest abundance was found in the cervical region of CE patients. In other studies, the overall abundance of mycoplasma, which often occurs in other studies, was very low. Only in the cluster thermogram analysis, we found the accumulation of mycoplasma in the cervical region of NP group. We believe that this may be related to the selection of patient groups. We select all patients who have undergone the early standardized treatment of the reproductive center and are ready to receive embryo transfer. Therefore, in the early stage of treatment, TCM students have already carried out systematic treatment for mycoplasma, so our results of mycoplasma are very low. Chlamydia only exists in the cervix and uterine cavity of CE, suggesting that Chlamydia may be related to the pathogenesis of CE.

## Conclusion

Our research results show that, compared with the normal control group, the endometrial microbiota of infertile patients with chronic endometritis or endometrial polyps has indeed changed significantly in the relative abundance distribution of species, which indicates that changes in local microecology may be an important factor in the occurrence of diseases or even adverse pregnancy outcomes. The further study of endometrial microecology may provide new opportunities for further improving the diagnosis and treatment strategies of chronic endometritis.

## Data availability statement

The data presented in the study are deposited in the BioProject (https://submit.ncbi.nlm.nih.gov/subs/bioproject/SUB12706297/overview) repository, accession number: BioProject ID: PRJNA932472.

## Ethics statement

The studies involving human participants were reviewed and approved by the ethics committee of Shanghai Tongji Hospital (SBKT-2022-036). The patients/participants provided their written informed consent to participate in this study.

## Author contributions

JL and LZ designed and carried out the study. YY and XJ collected samples and clinical data. ML and QZ conducted DNA extraction and PCR experiments. TL and ZH assisted in 16S rRNA sequencing. JL, QL, and XT analyzed data and wrote papers. LZ provides an important intellectual content revision for this article. All authors contributed to the article and approved the submitted version.
